# Some Remarks on the Pathology and Treatment of Dysmeorrhœa

**Published:** 1830

**Authors:** John Eberle


### WESTERN JOURNAL

OF THE

### MEDICAL AND PHYSICAL SCIENCES.

JULY, AUGUST, AND SEPTEMBER, 1830.

### ESSAYS AND CASES.

Art.—I. *Some Remarks on the Pathology and Treatment of Dysmenorrhœa.* By John Eberle, M. D., &c. &c.

The pathology of this very common and painful affection of females, does not appear to be well understood. Some writers, and among these Dr. Dewees, seem to think that this complaint depends invariably on the formation of a pseudo-membranous substance over the internal surface of the uterus, by which the orifices of the menstrual exhalents are obstructed. Such membranous structures are indeed frequently present in this disease, and they unquestionably impede menstruation where they do exist. It is nevertheless equally certain, I think, that difficult and painful menstruation, sometimes recurs without any such mechanical impediment. I have known young females invariably afflicted with extreme pain at each catamenial period for several years, without the discharge of any substance of this kind. It must moreover be observed that painful menstruation is by no means always attended with a scanty flow of the menstrual fluid. Burns makes this observation; and I have myself met with several remarkable instances of this kind.

Dr. Dewees observes that there are two distinct states of this affection. In one the mammæ sympathise with the uterus, and become tumid, and more or less painful. In the other no perceptible alteration in the state of the breasts occurs. The former of these varieties, he says is much more manageable than the latter, and this accords entirely with my own experience. The Dr. does not offer an explanation of this circumstance. It appears to me to admit of one. I have observed for instance, that in nearly every case where the breasts become tumid and painful, the concreted pseudo-membranous substance (if any is cast off) is thick and of much consistence; and in those where the mammæ do not sympathise, it is usually thrown off in the form of a thin membrane. In the former case, the cavity of the uterus is much more distended, approaching the condition of early pregnancy; and we may presume that this state would be most apt to awaken the uterine sympathies, and thus to excite the mammary glands. Such cases too are more readily removed, because much less expulsive effort is necessary to separate and expel a considerable mass, than a thin membraneous concretion adhering to the inner surface of the uterus.

Dr. Dewees' views concerning the pathology of this affection, appear to me not only contrary to sound pathological principles, but most unequivocally also to the import of its essential phenomena.

"In another place," "he says," I have declared, that the menstruous fluid is the product of a secretory process. I have there given my reasons for this opinion. I therefore now assume it as a principle; and upon this principle, attempt to account for the formation of the membranous production, so often yielded in dysmenorrhœa. But, before I attempt an explanation of the formation of this membrane, I must again direct attention to a very remarkable circumstance in the character of the menstrual blood, namely, its not possessing the property of coagulation. From this, it appears that the blood, or a part of it, has suffered some change by the action of the uterine vessels; and that this change has been imposed upon the coagulating lymph. by the process of secretion. I have assigned reasons for this change; when speaking of menstruation. Now, it is not difficult to suppose that the uterus, like every other organ, may have its functions impaired; in consequence of which the texture of the coagulating lymph, instead of being subdued as it is wont to be, when the uterine secretory action is perfect, it remains nearly the same as when it entered this viscus; except, that it may be attenuated, as in some inflammatory diseases: and it will from this imperfect elaboration, be thrown into the cavity of the uterus, without being dispossessed of the power of separation, and of coagulation. It is poured into the uterus in a very gradual manner; and, from this circumstance, may tarry there sufficiently long to separate into its constituent parts; the coloured part, or red globules, from their greater weight, will leave the imperfectly subdued coagulating lymph, and fall to the bottom of the uterus, and sooner or later be discharged; while the coagulating lymph, either in part or altogether, will be left to spread itself over the internal face of the uterus, and these quickly assume, as is usual with it when in contact with living parts, the appearance of a membrane."

From these observations, it appears that the Doctor considers a weak state of the uterus, or rather an impaired secretory function of this organ as the immediate cause of the production of the membrane in question. Unquestionably the menstrual action is deranged, but so far from this derangement, being the result of deficient uterine excitement, all the attending phenomena of the disease favor the idea that the excitement in the whole uterine system is morbidly increased, and that it approaches to the state of inflammation. The sense of fulness and pain in the pelvis, loins and thighs—the accelerated and often tense pulse—the hot and feverish skin, decidedly indicate a congested and irritated state of the pelvic organs. Analogy also affords us good grounds for this opinion. Lymph is never thrown out so as to form membranous concretions except from inflamed or highly irritated surfaces. The formation of such membraneous structures is indeed generally regarded as the most certain evidence of previous inflammation of the part upon which they appear. The opinion that dysmenorrhœa is a sub-inflammatory or highly irritated state of the internal surface of the womb, or of the uterus generally, is moreover supported by the fact that all *active or stimulating* emmenagogue remedies almost invariably, greatly aggravate the painful symptoms of the disease. Would this be the case if the disease depended on an impaired action of the uterine vessels? The general or constitutional habit apparently most favourable to the occurrence of this disease goes also to confirm the view I have taken of its character. It is seldom met with in debilitated, relaxed and phlegmatic habits. Robust irritable and sanguineous young females are most subject to it.

In consequence of impaired function of the secretory vessels, the blood, says Dr. Dewees, is thrown out with its power of coagulation and separation undiminished. In what then does it differ from ordinary hæmorrhage? In nothing, it would appear, according to his notion, except that "the lymph is perhaps somewhat attenuated as in some inflammatory diseases." But if membraneous encrustations of lymph arose from the slow effusion of coagulable blood, ought we not to meet with such structures in slow uterine hæmorrhages where the blood retains its power of coagulation and separation? This however does not occur.

I presume that the uterine vessels in this affection are much congested, and in a state of morbid irritability terminating in high irritation or subinflammatory action. The discharge at first flows for a short time, but the action of the secretory vessels soon transcends the grade of menstrual secretion, and instead of the regular catamenial fluid, lymph only is secreted by the irritated vessels, giving rise to the membranous structures in question.

From much attention to this affection, I am inclined to regard it as frequently of a rheumatic character. I have seen it alternate in two individuals with rheumatic pains in the joints of the inferior extremities, and I have succeeded frequently in removing it completely by the remedies deemed most effectual in rheumatic complaints. That rheumatism is apt to fix upon the uterus has been repeatedly observed. Cazenave states that he has frequently known rheumatic inflammation to fix upon the womb, and to give rise to very painful affections in this organ.* I have lately met with a striking instance of this kind. A young lady had been subject for many months to occasional pain and swelling of the knees, elbows, and wrists. Her menstrual functions continued regularly. Six months ago, she took flowers of sulphur to relieve her rheumatic affection. The articular pains and inflammation subsided, but she has since suffered extremely at each menstrual period, from the ordinary symptoms of dysmenorrhœa.

I shall not enter into a detail of the various remedies and modes of treatment that have been recommended in this affection. It may be sufficient to say, that the best palliative during the presence of the disease, so far as my own experience enables me to decide, is opium with camphor and ipecac. For the radical cure of this affection, Dr. Dewees recommends the tincture of guaiacum, and it is without doubt entitled to considerable attention as a remediate agent in this complaint. I have used it with success in a few cases, though I have been frequently disappointed with its employment. The remedy which I place most confidence in is the extract of the *stramonium*, or the tincture of the seed of this plant. I prescribed it at first on the presumption of the rheumatic character of the disease, and I have had much reason to be satisfied with its effects.

Miss A. M. aged 18, from the first appearance of the menses, regularly suffered the most severe pains at each menstrual period. A moderate discharge usually came on and continued only for a few hours. She was of a full habit, florid complexion, and in other respects in vigorous health. The pulse during each attack was accelerated, quick and tense ; her bowels were usually confined, and the stomach irritable. She was at first bled, and a saline purgative prescribed, with a simple and unirritating diet. The bleeding was repeated in about ten days and another dose of epsom salts administered. Eight days be-

*Memoir on the Treatment of Rheumatism.

fore the expected menstrual period, she began to take the extract of stramonium in quarter grain doses four times daily. This medicine was continued until slight vertigo ensued, which occurred on the third day. The menses appeared in a few days afterwards, but with much less suffering than formerly. During the ensuing interval, she was again bled to the extent of eight ounces and a dose of sulph. magnes. prescribed. Six days before the next menstrual period, she again took the stramonium as before. The menses ensued more copiously than they had ever done before, and with scarcely any suffering. By continuing the use of this narcotic, in the manner mentioned for four menstrual terms, the complaint was entirely subdued. A slight flocculent or membraneous substance was discharged at the third period after the use of this medicine. I could relate other instances which terminated equally favorably under the use of the stramonium. My friend Dr. M'Clintock, has used it with success in a case of this kind.

What would the tincture of colchicum do in this complaint? I have not used it; but its analogous powers with guaiacum and stramonium in rheumatic affections, justify the suspicion that it might be useful.

Mr. Patin, in a late No. of Rev. Medicale, has published some cases from which it would seem that the acetate of ammonia will often speedily suspend the excruciating pains of dysmenorrhœa—but more particularly those which attend carcinoma uteri. I have used it in this latter affection with manifest palliative effects.

